# Mullerian Cyst Presenting as an Inguinal Mass: A Rare Case Report

**DOI:** 10.7759/cureus.41209

**Published:** 2023-06-30

**Authors:** Nimah A Rabai, Arqam Alrababa, Saleh A Ba-Shammakh

**Affiliations:** 1 Department of General Surgery, Princess Basma Teaching Hospital, Irbid, JOR

**Keywords:** pax-8, wt1, inguinal, cyst, mullerian

## Abstract

Mullerian cysts are rare cystic lesions that represent remnants of Mullerian ducts. The clinical presentation usually involves swelling or symptoms of large cyst-size compression of adjacent structures. The preoperative diagnosis is very challenging due to the lack of specific features, and the precise diagnosis is reached with histopathological examination. In this report, we discuss a case involving a 26-year-old woman who visited our clinic with complaints of swelling in her left inguinal region. The patient was operated on as a case of a suspected left inguinal hernia, but the histopathological examination of the excised mass was consistent with the diagnosis of a Mullerian cyst.

## Introduction

The female uterus, fallopian tubes, and upper part of the vagina are formed during embryonic development by the fusion of the two Mullerian ducts [1]. Mullerian cysts are rare cystic lesions that develop from the remnants of Mullerian ducts. These cysts are typically benign and asymptomatic but can grow to a significant size that causes discomfort or pressure on surrounding tissues and organs [2]. Furthermore, Mullerian cysts are urogenital cysts reported in the pelvis of both males and females [3]. A definitive preoperative diagnosis is rarely possible; even during surgery, the true nature of Mullerian cysts may be difficult to identify by visual inspection [4]. Multiple imaging modalities help determine the cyst's location, size, and relation to the surrounding structure; however, the cyst’s characteristics cannot be judged [5]. Most of the time, the definitive identification of a Mullerian cyst relies on the results of histopathological examinations conducted after surgery. This article details the case of a 26-year-old woman who visited the general surgical outpatient clinic due to a noticeable swelling in her left inguinal region. The initial assessment pointed toward a left inguinal hernia.

## Case presentation

A 26-year-old female patient, married and with children, with a previous diagnosis of familial Mediterranean fever (FMF) and a surgical history of appendectomy and caesarean section (CS). The patient was seen in the outpatient clinic and had been complaining of left inguinal swelling for one year that was non-tender but increased in size with coughing. She was an infrequent smoker, not on any medications, including medications for FMF, and had a negative family history of malignancy. On examination, the patient had a thin built, looked healthy, and had normal vital signs. A mass measuring approximately 2.0 cm × 3.0 cm was seen in the left inguinal region with no overlying skin changes. On palpation, the mass was non-tender, with a positive cough impulse, and partially reducible. The impression was of a left inguinal hernia. Ultrasound (US) was done preoperatively; the differential diagnosis was a cystic lipoma versus a hernia containing bowel loops.

**Figure 1:**
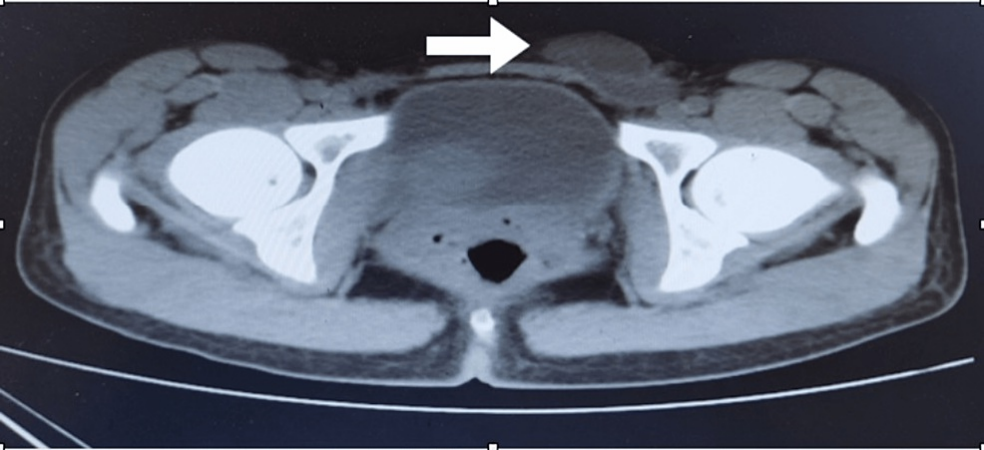
Abdominopelvic CT scan without contrast shows a lobulated hypodense lesion in the left inguinal area with connection to the deep inguinal region (arrow).

A computed tomography (CT) scan was done to confirm the diagnosis (Figure 1). It reported a well-defined lobulated oval-shaped hypodense mass lesion with a cystic component seen in the subcutaneous fat of the left inguinal region, measuring about 3.9 cm, with mildly faint surrounding fat stranding that appeared to be connected to the left deep inguinal region. These findings were suggestive of a left inguinal hernia, enlarged lymph nodes, or a mass lesion with a cystic component.

**Figure 2:**
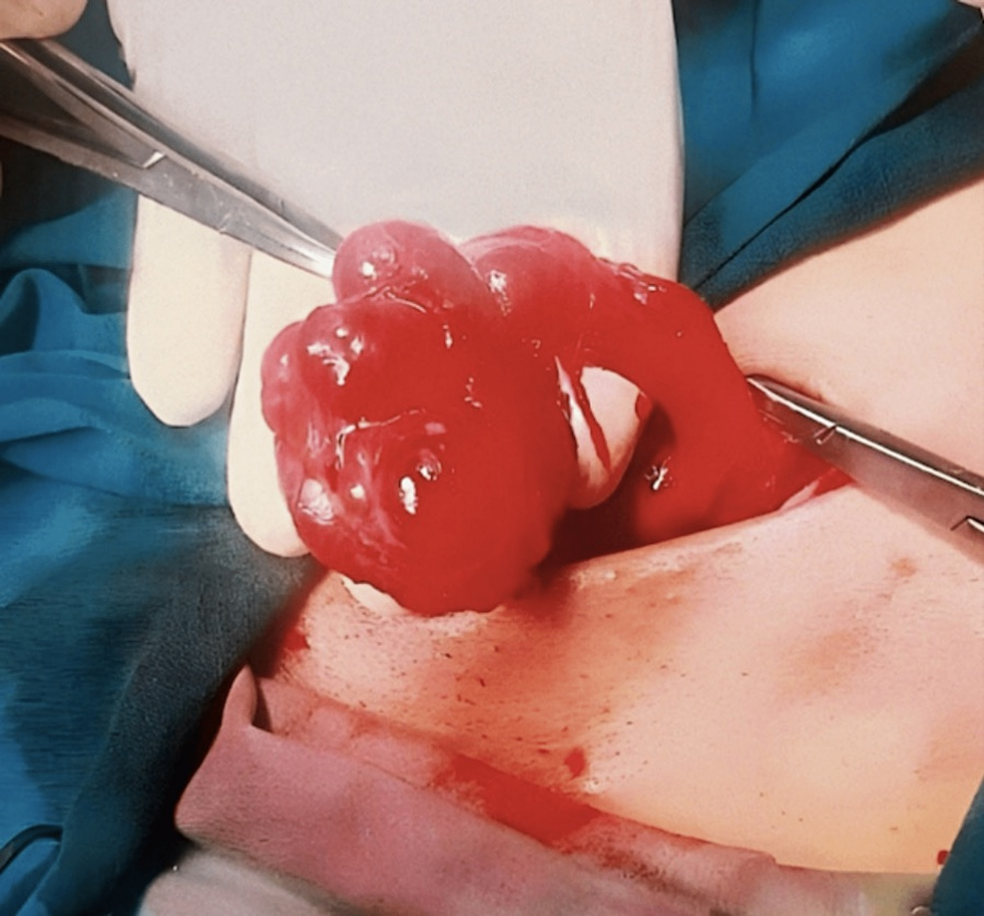
Intra-operative finding of a multicystic lesion.

Intra-operatively, a left inguinal incision was made and opened layer by layer down to the inguinal canal. After opening the inguinal canal, the mass was seen with multiple cysts and a thin strip of solid component, measuring approximately 4.0 cm × 2.0 cm × 1.5 cm (Figure 2). No other masses or hernia sacs were seen. Excision of the mass was performed and sent for histopathology.

The patient had an uneventful postoperative recovery and was discharged home the next day. No postoperative complications were reported during the subsequent follow-up visits. A gross histopathological examination showed an intact multilocular cystic structure measuring 4.0 cm × 2.0 cm × 1.5 cm, filled with brown fluid with no identifiable solid masses.

Microscopically, the cyst wall was lined by a single layer of polygonal cells with fine chromatin and clear cytoplasm, overlying an inflamed fibrofatty tissue with giant cell reactions and calcifications. No atypia or mitosis was identified. On immunohistochemistry, the lining cells were positive for WT1 and PAX-8, with variable positivity. The histopathological diagnosis was consistent with a Mullerian cyst with no evidence of malignancy.

## Discussion

Mullerian cysts usually occur in the body's midline, where their embryological site is located. In women, they have been seen around the uterus and ovary, where they may be misdiagnosed as primary ovarian or tubal cysts; they may also be seen from up there to the vagina, where they can present as vaginal wall cysts [6]. Some cases have been reported in men in the pelvis, primarily near the prostatic utricle [7]. In rare instances, Mullerian cysts have been documented outside of the typical pelvic and genitourinary organs, where they are classified into three groups: cutaneous ciliated cysts, retroperitoneal formations, and mediastinal Mullerian cysts. Diverse hypotheses have been advanced to account for their occurrence in non-typical locations: According to one hypothesis, the coelomic or peritoneal epithelium may differentiate into a serous or tubal-type epithelium. This then invaginates into the tissue beneath it, eventually severing its connection with the surface to create a cyst [8]. The theory of Mullerian heterotopia suggests that during the early stages of embryonic development (specifically the sixth to eighth week), remnants of Mullerian tissue endure a process of separation and migration [9].

These cysts range from less than 1 cm to large cysts that fill the pelvis and can hold up to 5 liters of fluid [10]. The size of our case was medium compared to the reported cases. The clinical presentation of these cysts depends on their location; most of them are asymptomatic. However, they can present as bulging, small masses on the lower abdomen or prolapsed vaginal masses [11]. Moreover, Mullerian cysts can present as abdominal discomfort, bloating, and fullness in the retroperitoneum when they become large enough to cause these symptoms [8].

The overall incidence of retroperitoneal cysts ranges from 1 in 5750 individuals to 1 in 250 000, with an average of 1 in 105 000. The patients are commonly of reproductive age and multiparous [12]. Interestingly, most cases of Mullerian cysts occur in obese women aged between 19 and 47 who receive hormonal therapy for menstrual irregularity [13]. Multiple studies showed that the hormonal effect should be considered an etiological factor for the growth of these cysts [12]. The patient presented here was thin-built and had no history of receiving hormonal therapy.

Computed tomography scans and magnetic resonance imaging (MRI) are commonly used in diagnosis. CT scans can detect unilocular or multilocular cysts with thin walls containing transparent fluid [13], whereas MRIs may exhibit low signal intensity in T1-weighted imaging and high signal intensity in T2-weighted imaging. However, pinpointing a preoperative diagnosis can be challenging due to the absence of distinctive clinical and radiographical characteristics [8].

Several therapeutic strategies, including external marsupialization, internal drainage, and straightforward aspiration, have been proposed. Even though these techniques are minimally invasive, they have been linked to high recurrence rates [14]. For the majority of patients, surgical excision remains an effective treatment. It is crucial that the entire cyst be removed during the procedure, as leaving any portion of a multilocular cyst may result in a recurrence [4].

Because primary adenocarcinoma can develop from a Mullerian cyst, the presence of malignancy cannot be ignored in these cases [15]. A definitive diagnosis is made by taking a specimen for histopathologic study. On histopathological examination, the lining of these lesions is predominantly composed of mucinous epithelium. However, they may also have a lining derived from any epithelium with Mullerian origins, such as the endocervix, endometrium, or fallopian tubes [6]. A rare yet important differential diagnosis is pseudomyxoma peritonei. Around 4% of female patients with pseudomyxoma peritonei present with an inguinal hernia [16]. Therefore, the role of immunohistochemistry becomes pertinent. On immunohistochemistry, the lining of Mullerian cysts is usually positive for hormonal receptors like estrogen and progesterone receptors and positive for the immunohistochemical stains PAX-8 and WT1 [17]. In our reported case here, the cystic wall cells were variably positive to PAX-8 and WT1 stains, which was consistent with the diagnosis of Mullerian cyst, and there was no evidence of malignancy or recurrence that has been reported till now over a year of follow-up.

## Conclusions

Despite their rarity, it is essential to consider Mullerian cysts when diagnosing inguinal cystic lesions. The histopathological examination is the only confirmatory diagnostic test, especially to rule out any evidence of primary malignancy, therefore allowing physicians to provide proper management and follow-up to patients with this pathology.
